# Neurological complications of COVID-19: a preliminary review

**DOI:** 10.1007/s00415-020-09941-x

**Published:** 2020-06-03

**Authors:** A. Pryce-Roberts, M. Talaei, N. P. Robertson

**Affiliations:** Division of Psychological Medicine and Clinical Neuroscience, Department of Neurology, Cardiff University, University Hospital of Wales, Heath Park, Cardiff, CF14 4XN UK

## Introduction

Coronavirus disease 19 (COVID-19) is a highly transmissible viral infection caused by severe acute respiratory syndrome coronavirus 2 (SARS-CoV-2), which emerged in Wuhan, China and has since spread rapidly around the world. Coronaviruses are so named for their characteristic crown-like appearance and are widespread in nature infecting several different species causing mainly respiratory and enteric pathology [1].

SARS-CoV-2 is the seventh coronavirus known to infect humans; whereas four are endemic (HKU1, NL63, OCw43 and 229E) and cause a considerable share of mild upper and lower respiratory tract infections, SARS-CoV, MERSCoV and SARS-CoV-2 can cause severe disease [2]. The spectrum of pathology in COVID-19 is wide and ever increasing with a burgeoning number of case reports and case series published on a daily basis that provide some preliminary insights to the disease and its complications. However, it is certain that larger case series and population-based studies will follow, as is the usual pattern with novel diseases. In this month’s journal club, and in a break from the normal format, we have attempted to provide an early snapshot of the current literature describing neurological manifestations of this novel disease in a very fast moving subject area, but in the certain knowledge that this will be superseded in due course by more comprehensive studies.

## Neurological manifestations of COVID-19

As of the 1st May 2020, there have been 52 data papers comprising case reports or case series and 27 reviews including editorials/position papers. In January 2020, a retrospective, single-center study on 99 patients diagnosed with SARS-COV-2 pneumonia at a hospital in Wuhan identified confusion and headache in 9% and 8%, respectively [3]. A recently published retrospective, observational study from Wuhan in 214 patients with RT-pPCR confirmed COVID-19 reported 36% had neurologic manifestations [4]; central nervous system (CNS) 25%, peripheral nervous system (PNS) 9% and skeletal muscle injury 11%. In patients with CNS manifestations, the most commonly reported symptoms were dizziness (17%) and headache (13%). In patients with PNS symptoms, the commonest were dysgeusia 6% and hyposmia 5%. CNS symptoms were also more commonly identified in those with severe disease with associated thrombocytopenia, increased urea levels and lymphopenia.

A study of patients admitted to intensive care units (ITU) has also emphasized the frequency of neurological features [5]. In an observational series of 58 of 64 consecutive patients admitted to hospital with acute respiratory distress syndrome (ARDS) secondary to COVID-19, 40 (69%) displayed agitation and 39 (67%) exhibited diffuse corticospinal tract signs with enhanced tendon reflexes, ankle clonus, and bilateral extensor plantar reflexes. Of the patients who had been discharged by the time of publication, 15/45 (33%) were noted to have a dysexecutive syndrome consisting of inattention, disorientation, or poorly organized movements in response to command. However, in the seven patients who underwent CSF analysis, RT-PCR for SARS-CoV-2 was negative in all cases.

In addition to neurological symptoms accompanying the classical respiratory presentation of COVID-19, case reports and series have identified a wide range of acute clinical neurological syndromes which largely fall into three main categories: stroke, Guillain–Barré Syndrome (GBS) and variants and meningoencephalitis/encephalopathy/encephalitis. There are also a smaller numbers of studies reporting epilepsy (*n* = 3), acute disseminated encephalomyelitis (ADEM) and acute myelitis. In addition there are reports of psychiatric and neuropsychiatric presentations which are not reviewed in this article.

## Stroke

Of 12 reports published up to the 1st May 2020, 5 were single case reports, 4 were case series (reporting between 2 and 6 cases), 2 were observational studies describing the incidence of CVA in patients positive for RT-PCR for SARS-CoV-2, whilst a further study is a meta-analysis of four studies which reported that patients with a history of stroke have an increased odds ratio of 2.5 for developing severe COVID-19 [6]. The two observational studies describe case series of patients with COVID-19. In the first retrospective study of 221 patients, 11 (5%) developed acute ischemic stroke, 1 (0·5%) cerebral venous sinus thrombosis (CVST), and 1 (0·5%) cerebral hemorrhage [7]. In the second prospective study of 288 patients, ischemic stroke was diagnosed in 9 (2.5%) of included patients [8].

These 9 case reports and case series together detail a total of 21 patients; [9–17] 16 males and 5 females. Age was precisely stated in 20 cases and was a mean of 59.8 years (male-63.1, female-49.8), although may be artificially depressed by the case series of young strokes [12]. The majority (19) were ischemic strokes, although three underwent hemorrhagic transformation, whilst 2 were hemorrhagic at onset. Of the 19 ischemic cases, 8 had multiple infarcts. Thirteen patients had thrombus detectable on imaging; 6 within the middle cerebral, 2 posterior cerebral, three vertebral/basilar and 1 internal carotid. A further case had multiple sites of thrombus. Of the remainder, four were consistent with large-vessel occlusions but the presence of thrombus was not explicitly stated; the remaining two were lacunar infarcts. Several of the reports note other thrombotic events; four patients had pulmonary emboli (three bilateral), one patient presented with myocardial infarction and ischemic limbs, while ischemic limbs and renal infarction were also noted in other cases. Of the 19 patients with ischemic stroke, four patients died, six were still supported on an intensive care unit (ICU), one remained on a stroke unit, two were at a stroke rehabilitation facility and five made a good recovery. Outcome in one case was not stated.

Also of interest, significantly elevated D-Dimer levels (≥ 1000 μg/L) were reported in 14 cases, of which nine had anticardiolipin antibodies and lupus anticoagulant tests with variable results. In a series of six patients from the UK, one patient had medium titre for IgM anticardiolipin antibodies and lupus anticoagulant was positive in five of six cases [10]. However in a series of three patients from Beijing, all patients were positive for anticardiolipin IgA antibodies as well as anti-β2-glycoprotein I IgA and IgG antibodies but lupus anticoagulant was not detected in any patients [13].

## Guillain-Barré Syndrome and variants

Of 8 reports published by 1st of May 2020, 6 were single case reports [18–23] and 2 were case series (*n* = 2–5) [24, 25] describing a total of 13 para-COVID-19 or post COVID-19 GBS (10 males, 3 females, mean age 58.9; male 55.8, female 69.7). Eleven were positive for SARS-COV-2 RT-qPCR from oropharyngeal swabs. Two were RT-qPCR negative; one presented 24 days after COVID-19 respiratory symptoms but with typical CT chest abnormalities [19] and one where the GBS presentation preceded respiratory symptoms by 8 days [23].

Eleven cases presented with progressive a flexic flaccid tetraparesis of whom five developed bilateral facial weakness and seven required intubation and ventilation. Two cases presented with a Miller Fisher variant [25]. Neurological symptoms developed between 3 and 28 days after emergence of respiratory symptoms at a median interval of 11 days. Twelve of the 13 cases were treated with IVIG. At 4 weeks, two patients required ongoing mechanical ventilation in ITU, three were undergoing rehabilitation, four had been discharged and one had died. Outcomes for the remaining three patients are unknown.

Lumbar puncture was performed on 11 patients from day 1 to 5 of neurological symptoms; white cells were typically normal or mildly raised (1–9/μL in 5 cases), protein was within normal range in 3 cases and > 45 mg/dL in the remaining 8 cases (range 48–193 mg/dL; average 104.6 mg/dL). Eight had CSF tested for SARS-COV2 PCR all of which were negative. Ten had neurophysiological examinations between day 3 and 12 of neurological symptoms; 6 showed changes consistent with demyelination and 4 with axonal variant GBS. Anti-ganglioside antibodies results were reported in only four; three were negative whilst a patient with Miller Fisher presentation had positive GD1b antibody but negative GQ1b [25].

## Encephalitis, meningoencephalitis and encephalopathy

Of the 8 reports published up to May 1st 2020, 7 were single case reports [26–32] and one a case series (*n* = 2) [33] in the context of oropharyngeal swabs or CSF positive for SARS-Cov-2 using RT-qPCR. Of the 9 cases, 5 were females and 3 were males; one did not specify gender; average age 55.5; male 42.0, female 60.8. Clinical presentations were generally consistent with the fever and confusion commonly described. Meningism was noted in three, headache in four, seizures in three and three exhibited frontal release signs. A single case of acute necrotizing encephalopathy in a confirmed COVID-19 patient was also reported [28].

Imaging failed to demonstrate acute changes in the majority. However, in one case hyperintense signal in the right mesial temporal lobe and hippocampus was noted [30] whilst hemorrhagic rim enhancing lesions within the bilateral thalami, medial temporal lobes and subinsular regions was reported in the case of acute necrotizing encephalopathy [28]. EEG was performed in two of the cases presenting with seizures as well as one other with encephalopathy [29]; all showed generalized slowing.

Of the nine published cases, seven tested CSF for SARS-Cov-2; two noted that CSF testing was not available at their institution [28, 31]. Two cases had positive CSF PCR for SARS-Cov-2 [26, 30], five were negative [27, 29, 32, 33]. The majority of patients were treated with broad spectrum antibiotics and antiviral medications. Two recovered spontaneously within 4 days [33] and one showed little response to antivirals and hydroxychloroquine but appeared to make a dramatic recovery on commencement of high dose steroids and was discharged within 2 weeks of presentation [29]. Two others who showed little response to initial treatment improved with a mannitol infusion [27] and hydroxychloroquine [31], respectively. Two patients remained in ITU [30, 32] and no clear outcome was documented in a further two cases [26, 28].

## Comment

It is becoming apparent that neurological complications of COVID-19 are relatively common together with a suggestion that they are seen more frequently with increasing disease severity. In common with previous pandemics including Zika and SARS-CoV, stroke, GBS and meningoencephalitis are emerging as the commonest specific neurological complications but causality remains difficult to prove. Stroke in particular may have a plausible biological mechanism whilst GBS and meningoencephalitis have a compelling temporal relationship and are likely to represent parainfectious phenomenon. However, whether they are specific to COVID-19 or whether they simply represent the consequence of a burden of infection remains unclear. In addition cognitive impairment is also emerging as a common sequelae in COVID-19 patients discharged from ITU and we can expect an increase in referrals to memory clinics in the short to medium term. Only longer term epidemiological studies will clarify some of these issues and will need to be supported by research on the neurotropic nature of the virus and biological mechanisms.
